# Polymorphic Single‐Nucleotide Variants in miRNA Genes and the Susceptibility to Colorectal Cancer: Combined Evaluation by Pairwise and Network Meta‐Analysis, Thakkinstian's Algorithm and FPRP Criterium

**DOI:** 10.1002/cam4.70621

**Published:** 2025-01-22

**Authors:** Qing Liu, Ivan Archilla, Sandra Lopez‐Prades, Ferran Torres, Jordi Camps, Miriam Cuatrecasas

**Affiliations:** ^1^ Faculty of Medicine and Health Sciences, Doctoral School University of Barcelona Barcelona Spain; ^2^ August Pi i Sunyer Biomedical Research Institute (IDIBAPS) Barcelona Spain; ^3^ Pathology Department, Centre of Biomedical Diagnosis (CDB) Hospital Clinic, University of Barcelona Barcelona Spain; ^4^ Department of Biostatistics Autonomous University of Barcelona (UAB) Bellaterra Spain; ^5^ Centro de Investigación Biomédica en Red en Enfermedades Hepáticas y Digestivas (CIBEREHD) Madrid Spain; ^6^ Cell Biology and Medical Genetics Unit, Department of Cell Biology, Physiology and Immunology, Faculty of Medicine Autonomous University of Barcelona (UAB) Bellaterra Spain; ^7^ Faculty of Medicine and Health Sciences, Department of Clinical Foundations University of Barcelona (UB) Barcelona Spain

**Keywords:** colorectal cancer, false positive report probability, microRNA, network meta‐analysis, single‐nucleotide polymorphism, susceptibility, Thakkinstian's algorithm

## Abstract

**Background:**

Considerable epidemiological studies have examined the correlation between polymorphic single‐nucleotide variants (SNPs) in miRNA genes and colorectal carcinoma (CRC) risk, yielding inconsistent results. Herein, we sought to systematically investigate the association between miRNA‐SNPs and CRC susceptibility by combined evaluation using pairwise and network meta‐analysis, the FPRP analysis (false positive report probability), and the Thakkinstian's algorithm.

**Methods:**

The MEDLINE, EMBASE, WOS, and Cochrane Library databases were searched through May 2024 to find relevant association literatures. Pooled odds ratios (ORs) and 95% confidence intervals (CIs) were computed by the pairwise meta‐analysis. Network meta‐analysis and the Thakkinstian's method were applied for determining the potentially optimal genetic models; additionally, the FPRP was used to identify noteworthy associations.

**Results:**

Totally, 39 case–control trials involving 18,028 CRC cases, and 21,816 normal participants were included in the study. Eleven SNPs within nine genes were examined for their predisposition to CRC. miR‐27a (rs895819) was found to significantly increase CRC risk among overall population (OR 1.58, 95% CI: 1.32–1.89) and Asians (OR 1.62, 95% CI: 1.31–2.01), with the recessive models identified as the optimal models. Furthermore, miR‐196a2 (rs11614913), miR‐143/145 (rs41291957), and miR‐34b/c (rs4938723) were significantly related to reduced CRC risk among Asian descendants under the optimal dominant (OR 0.75, 95% CI: 0.65–0.86), recessive (OR 0.72, 95% CI: 0.60–0.85), and recessive models (OR 0.69, 95% CI: 0.56–0.85), respectively. The results were also proposed by the network meta‐analysis or the Thakkinstian's method and confirmed by the FPRP criterion.

**Conclusion:**

The miR‐27a (rs895819) is correlated with elevated CRC risk among overall population and Asians, and the recessive model is found to be optimal for predicting CRC risk. Additionally, the miR‐196a2 (rs11614913), miR‐143/145 (rs41291957), and miR‐34b/c (rs4938723), with the dominant, recessive, and recessive models identified as the optimal, might confer protective effects against CRC among Asians.

## 1 Introduction

In 2022, GLOBOCAN estimated that colorectal cancer (CRC) ranked the 3rd most prevalent carcinoma with 1,926,425 newly diagnosed cases globally, being the 2nd most lethal malignancy [1]. In fact, CRC represents about 9.6% of all newly diagnosed cancer cases and accounts for approximately 9.3% of deaths due to cancer [1]. Genetic mutations are crucial in the CRC carcinogenesis. Based on genetic background, CRC can be categorized into three subsets: hereditary, familial, and sporadic CRC [2]. About 1/3 CRC cases exhibit familial clustering; however, merely 5%–16% of patients are related to a germline variant in genes that predispose individuals to CRC [3]. Unlike hereditary CRC being mostly attributed to specific and precise high‐penetrance mutations, sporadic CRC, which accounts for 60%–80% of CRCs, mainly involves low‐penetrance mutations [4, 5]. The etiology of sporadic CRC involves a multifaceted disorder which includes genomic alterations, environmental factors, and gene/environmental interactions [6, 7, 8, 9, 10, 11].

The secondary strategy for CRC prevention is based on screening tests. Previous studies have shown that screening greatly enhances early detection and expedite surgical treatment, and is associated with a more minimally invasive surgery, reduced complications and emergent surgeries; ultimately, all these contributed to enhancements in both overall survival and disease‐free survival [12, 13]. The most often utilized screening tests include fecal immunochemical test (FIT), fecal occult blood testing (FOBT), and colonoscopy. As an invasive assay, colonoscopy is not well accepted by eligible individuals for CRC screening. Yet, FOBT and FIT have medium patient compliance and some false positive detection rate [14, 15]. Nevertheless, most patients with FOBT and FIT positive results undergo colonoscopy, which is an invasive procedure. Thus, there is an unmet need for predicting CRC susceptibility with considerable accuracy and high patient compliance, which can be incorporated into routine clinical workup.

MicroRNAs (miRNAs) are endogenous, short, non‐coding and single‐stranded sequences comprising 18–25 nucleotides, which capable of targeting on mRNAs' complementarity sequences for regulating post‐transcriptionally their functions as oncogenes or tumor suppressors [16, 17]. miRNAs have an impact on almost all biological processes, including angiogenesis, proliferation, apoptosis, and invasion/metastasis [18]. Approximately 50% of miRNAs are situated in fragile locations and susceptibility regions [19]. Single‐nucleotide polymorphisms (SNPs) are the most prevalent genetic variation in the human genome, occurring around once per 300 nucleotides and accounting for more than 90% of all variations between individuals [20]. SNPs mapping in the miRNA promoter locus or seed sequence can influence miRNA transcription, maturation, and its interactions with mRNA [21, 22]. The regulatory mechanisms governing miRNAs expression are closely related to cancer diagnosis, treatment and prognosis, as well as the pathogenesis [17]. The alteration of function in specific miRNAs appears to be a pivotal event in the development of various malignancies [22]. In fact, SNPs in miRNA genes can serve as biomarkers, not only for predicting cancer susceptibility, but also treatment response [23, 24]. Currently, many miRNA‐SNPs have been identified to relate to CRC risk by genetic association analyses. Circulating miRNA in body fluids including human milk, tears, serum, saliva, seminal fluid, urine, and feces, can be detected and may serve as potential non‐invasive diagnostic biomarkers for CRC [22, 25].

Numerous epidemiological studies have examined the association between miRNAs‐SNPs with CRC risk, yielding inconsistent findings. Multiple meta‐analyses have also been conducted concerning the impact of miRNA‐SNP on CRC risk; however, most of the studies focused on a single or a limited number of miRNA‐SNPs. To date, only three studies have provided a field synopsis of SNPs in association with CRC risk [26, 27, 28]. Furthermore, the outcomes of these meta‐analyses remain contradictory and unconvincing due to limited sample size and vast heterogeneities attributable to various ethnicity and cancer type of individuals included. The present study will pinpoint the most relevant miRNA‐SNPs that affect the CRC development. We aimed at identifying the optimal genetic models by network meta‐analysis and the Thakkinstian algorithm [29]. As the initial statistically significant findings of associations between SNPs and cancer might be false positive, we also assessed the false positive report probability (FPRP) to decide whether the result is noteworthy [30]. As far as we know, we are the first to investigate the association between miRNA‐SNPs and CRC susceptibility using a combined approach of pairwise and network meta‐analysis, the Thakkinstian's method, and the FPRP method.

## 2 Methods

### 2.1 Article Search and Selection Criteria

Studies published up to May 2024 that investigated frequency differences in miRNA‐SNPs between sporadic CRC cases and normal participants were screened by searching the MEDLINE, WOS, EMBASE, and Cochrane Library databases. Only human studies published in English or Chinese were included. The studies in Chinese, which only the abstracts were included by abovementioned databases, were obtained in full text by searching the China National Knowledge Infrastructure (CNKI) or Wanfang databases. No regional restriction was applied. The search techniques comprised Medical Subject Headings and free text terms including the following: “Colorectal Neoplasm,” “Colorectal cancer,” “Single Nucleotide Polymorphism,” “SNP,” “MicroRNA,” “miRNA.” The Data S1 showed the details of our search strategy. The reference lists of all included studies were checked meticulously for additional eligible literatures.

To find the eligible studies, the following criteria for inclusion were adopted: (1) full‐text case–control studies comparing frequency variations in miRNA‐SNPs between sporadic CRC cases and healthy participants; (2) primary CRC diagnosed by histopathological examination; (3) sufficient information including the genotype allele frequency were provided; and (4) healthy controls had no malignant diseases or cancer histories. Studies were excluded for the following reasons: (1) case reports, series, reviews, abstract only, comment, editorials, conference report, or animal research; (2) duplicate data; (3) non‐English or Chinese articles; (4) studies which had the controls violating the Hardy–Weinberg equilibrium (HWE) for SNPs; (5) miRNA‐SNPs that were reported less than twice among all included studies; (6) total sample size less than 300 individuals; (7) studies with high risk of bias in quality assessment (total score < 7); and (8) contradictory or incredible outcomes reported. In addition, no limitations were imposed on age, sex, ethnicity, country of origin, genotype detection method, or cancer stage.

### 2.2 Study Selection and Data Extraction

**FIGURE 1:**
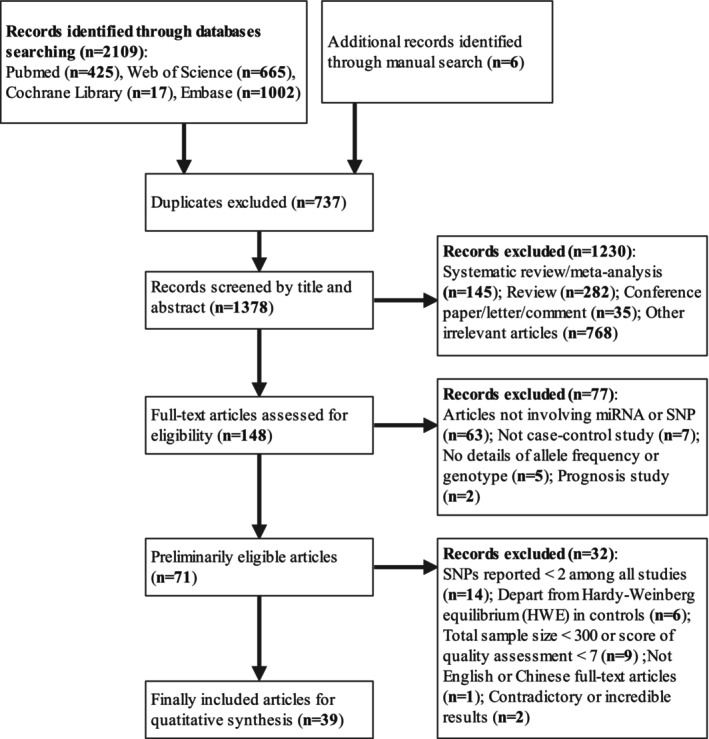
PRISMA flow diagram of literature searching for the meta‐analysis.

Two authors (Q.L., I.A.) were responsible for study inclusion and extracting the relevant data, with discrepancies resolved by discussion. Totally, 2,109 articles were identified through the databases searching. Additionally, six articles were obtained by manually searching. After excluding 737 duplicates, 1,78 records were examined by reading titles and abstracts, then 148 full‐text articles were assessed for eligibility. Finally, 39 eligible studies were included for quantitative synthesis (Figure 1). Data extracted from all the included studies are listed as follows: last name of the first author, publication year, country, sample size, source of controls (population‐ or hospital‐based), case–control matching, ethnicity, sex, age at diagnosis, type of miRNA‐SNPs, genotyping techniques, frequencies of genotypes, and HWE. In addition, minor allele frequency (MAF) for controls was also extracted or calculated if not provided.

### 2.3 Methodological Quality Assessment of Studies

The assessments of all studies were done based on a pre‐established rating scale (Table 2). The detailed items for evaluation were listed in Data S2. The assessment involved seven domains in total: (1) representativeness of case; (2) representativeness of control; (3) ascertainment of cancer; (4) case–control matching; (5) genotyping examination; (6) deviation from HWE; and (7) total sample size. The studies were successively appraised and scored hierarchically from a sequence of low to high risk of bias within each domain [(N_(item)_‐1) ~ 0]. Totally, a 15‐point scale was used for the quality assessment; studies were defined as high quality (low risk of bias) if they had scores ≥ 11, medium quality (intermediate or unknown risk of bias) with 7 ~ 10 scores, poor quality (high risk of bias) for scores < 7 and in this case, they were excluded. Two reviewers (Q.L., I.A.) conducted the assessment independently, and discrepancies were addressed by debate or referring to another author (J.C.).

### 2.4 Statistical Analysis

For pairwise meta‐analysis, pooled odds ratios (ORs) with 95% confidence intervals (CIs) were calculated under six genetic models (allelic, homozygous, heterozygous, dominant, recessive, and over‐dominant models). If *p* > 0.1 (*Q* test) with *I* ^2^ < 50%, indicating the absence of heterogeneity, a fixed effects model was used. Otherwise, if *p* < 0.1 (Q test) or *I* ^2^ > 50%, a random effects model was applied. A leave‐one‐out analysis was done by repeatedly removing one study to ensure that our findings were not driven by any individual study. Subgroup analyses and meta‐regressions were performed according to ethnicity (Caucasian, Asian) and genotypic method (RFLP‐PCR, TaqMan, and Others) to further examine the sources of heterogeneity. Publication bias was evaluated using Begg's and Egger's tests, other than in cases that fewer than 10 studies were included. A random‐effects network meta‐analysis was performed within a Bayesian framework utilizing the R packages “gemtc.” The four parallel Markov chain Monte Carlo (MCMC) simulations were run for a burn‐in phase of 20,000 iterations, followed by an extra phase of 50,000 iterations. The convergence was assessed using potential scale reduction factor (PSRF), with a PSRF around 1 signifying a convergent model. The surface under the cumulative ranking curve (SUCRA) scores were used to rank the probabilities of genetic models of the miRNA‐SNPs. The genetic models of miRNA‐SNPs with larger SUCRA scores are considered to be more suitable genetic models for predicting CRC risk.

The Thakkinstian's method was also utilized to identify the potentially optimal model [29]. A SNP comprises a dominant allele (A) and a recessive allele (a). By meta‐analysis, AA versus aa (OR1), Aa vs aa (OR2) and AA versus Aa (OR3) along with their corresponding 95% CIs were calculated, respectively. The comparisons among these ORs can help to select the potentially optimal genetic model. When OR1 = OR3 ≠ 1 and OR2 = 1, a recessive model is proposed. When OR1 = OR2 ≠ 1 and OR3 = 1, a dominant model is suggested. When OR2 = 1/OR3 ≠ 1 and OR1 = 1, a complete over‐dominant model is proposed. When OR1 > OR2 > 1 and OR1 > OR3 > 1 (or OR1 < OR2 < 1 and OR1 < OR3 < 1), then a codominant model is suggested [29].

To evaluate the noteworthiness of the candidate genetic models associated with CRC risk, the false positive report probability (FPRP) was calculated by a predetermined Excel spreadsheet under five levels of prior probabilities (0.1, 0.01, 0.001, 0.0001, and 0.00001) and an OR of 1.5 [30]. In the present study, the criteria for assessing the optimal genetic model are as follows: (i) a minimum of two studies were needed for the assessment of potentially optimal models, (ii) the potentially most appropriate genetic models were determined by network meta‐analysis or Thakkinstian's method or both, (iii) noteworthy results by FPRP analysis (by setting an OR of 1.5, FPRP value < 0.2 under the prior probabilities of below 0.01) were obtained. In case that two genetic models of one SNP meet all the criteria, they will all be regarded as optimal models.

The diagnostic meta‐analysis was conducted to assess the diagnostic performance of the optimal genetic model predisposing to CRC by the Meta‐DiSc software [31]. Pooled sensitivity, specificity, positive likelihood ratio (+LR), negative likelihood ratio (−LR) and diagnostic odds ratio (DOR) were calculated. Furthermore, summary receiver operating characteristic (SROC) curve with the area under the curve (AUC) was also illustrated. Spearman correlation analysis was employed to assess heterogeneity associated with threshold effect.

All statistical analyses were conducted by R 4.2.2 (https://www. R‐project.org/) and Meta‐DiSc 1.4 [31]. The forest plot was generated by Stata 17.0 (https://www.stata.com/). The test for statistical significance was set at *p* < 0.05 (*p* < 0.1 for heterogeneity).

## 3 Results

### 3.1 Study Characteristics

The procedure for literature selection is shown in Figure 1. Totally, 39 case–control studies comprising 18,028 CRC cases and 21,816 healthy participants were included for quantitatively investigating the association of miRNA‐SNPs with CRC susceptibility using meta‐analyses [32, 33, 34, 35, 36, 37, 38, 39, 40, 41, 42, 43, 44, 45, 46, 47, 48, 49, 50, 51, 52, 53, 54, 55, 56, 57, 58, 59, 60, 61, 62, 63, 64, 65, 66, 67, 68, 69, 70]. Eleven SNPs across nine genes were evaluated in these studies (Table 1). These studies were published from 2011 to 2023, with sample sizes varying between 313 and 2,429 individuals. Of all studies, 28 were based on Asian descendants while the remaining 11 were based on Caucasian descendants; 14 involved miR‐196a2 (rs11614913), 17 involved miR‐146a (rs2910164), seven involved miR‐27a (rs895819), four involved miR‐499 (rs3746444), four involved miR‐149 (rs2292832), three involved miR‐608 (rs4919510), five involved miR‐143/145 (rs41291957), two involved miR‐143/145 (rs353292), two involved miR‐143/145 (rs4705341), three involved miR‐34b/c (rs4938723), and two involved miR‐124 (rs531564) (Table 1). The preferred methods for genotyping were RFLP‐PCR, used in 19 studies, and TaqMan, used in 13 studies, among others. The detailed information of the studies is available in Data S3. The controls in all the studies had miRNA‐SNPs that adhered to HWE. The methodological assessment of the trials is shown in Table 2. It shows that 15 studies were classified as high quality with a low risk of bias, while 24 others were deemed as medium quality for intermediate or unknown risk of bias.

**TABLE 1:** Selected SNPs with corresponding articles.

Labels	Genes	SNPs
A	miR‐196a2	rs11614913 [32, 33, 39, 41, 47, 49, 51, 52, 53, 54, 55, 57, 58, 62]
B	miR‐146a	rs2910164 [35, 37, 41, 43, 44, 48, 49, 51, 52, 53, 54, 55, 58, 59, 60, 61, 68]
C	miR‐27a	rs895819 [42, 46, 53, 54, 56, 58, 69]
D	miR‐499	rs3746444 [43, 44, 49, 52]
E	miR‐149	rs2292832 [49, 50, 51, 55]
F	miR‐608	rs4919510 [58, 61, 67]
G	miR‐143/145	rs41291957 [40, 45, 61, 64, 70]
H	miR‐143/145	rs353292 [34, 64]
I	miR‐143/145	rs4705341 [38, 45]
J	miR‐34b/c	rs4938723 [36, 63, 65]
K	miR‐124	rs531564 [61, 66]

**TABLE 2:** Quality assessment of the included studies.

Low risk of bias	
Intermediate or unknown risk of bias	
High risk of bias	

Studies	Representativeness of case	Representativeness of control	Ascertainment of cancer	Case–control matching	Genotyping examination	HWE^a^	Total sample size	Total scores
Zhan, 2011	1	1	2	2	2	1	2	11
Chen, 2012	1	1	2	2	1	1	2	10
Hezova, 2012	1	1	2	1	0	1	1	7
Min, 2012	1	3	2	2	1	1	2	12
Zhang, 2012	2	3	2	2	1	1	2	13
Zhu, 2012	1	1	2	2	2	1	3	12
Ryan, 2012	1	2	2	1	2	1	2	11
Chae, 2013	1	3	2	2	1	1	2	12
Gao, 2013	1	1	2	2	2	1	2	11
Ma, 2013	1	3	2	2	2	1	3	14
Vinci, 2013	1	1	2	2	1	1	1	9
Lv, 2013	1	1	2	1	1	0	2	8
Li, 2013	1	1	2	0	1	1	2	8
Cao, 2014	1	1	2	2	0	1	1	8
Hu, 2014	1	1	2	2	1	1	2	10
Kupcinskas, 2014	1	1	2	0	1	1	2	8
Mao, 2014	2	3	2	2	2	1	3	15
Oh, 2014	1	3	1	1	1	1	2	10
Parlayan, 2014	1	1	2	0	2	1	2	9
Bian, 2015	1	1	2	2	1	1	2	10
Dikaiakos, 2015	1	1	2	2	0	1	1	8
Gao, 2015	1	1	2	2	0	1	3	10
Jiang, 2016	1	1	2	2	1	1	3	11
Ying, 2016	1	3	2	2	1	1	3	13
Sun, 2016	1	1	2	1	2	1	3	11
Yuan, 2016	1	1	2	1	1	1	3	10
Chayeb, 2018	1	1	2	2	2	1	1	10
Gao, 2018	1	1	2	2	1	1	3	11
Haerian, 2018	1	1	2	2	0	1	3	10
Kassim, 2019	1	3	2	2	1	1	3	13
Santos, 2020	1	1	2	1	0	1	1	7
Ahmed, 2020	1	1	2	2	1	0	1	8
Zhang, 2020	1	1	2	2	1	1	2	10
Soltanian, 2021	1	1	2	0	1	1	1	7
Jiang, 2021	1	1	2	2	1	1	3	11
Wang, 2021	1	1	2	1	2	1	2	10
Trujillo, 2023	1	1	2	2	1	1	1	9
Feng, 2023	1	1	2	2	1	1	2	10
Yueh, 2023	1	1	2	2	0	1	2	9

*Note:* The detailed items for the assessment were listed in Data S2.

Abbreviation: HWE, Hardy–Weinberg equilibrium.

^a^ In studies (Lv 2013 and Ahmed 2020), which contained SNPs violating HWE, we only included the SNPs which conformed to HWE.

### 3.2 Pairwise Meta‐Analysis

For miR‐196a2 (rs11614913), our findings indicated that TT and TC genotypes significantly lowered CRC risk compared to CC genotype (OR 0.85, 95% CI: 0.74–0.99). For miR‐27a (rs895819), all genetic models excluding the heterozygote and over‐dominant models predisposed individuals to CRC (allelic: OR 1.22, 95% CI: 1.11–1.33; homozygote: OR 1.62, 95% CI: 1.34–1.96; dominant: OR 1.15, 95% CI: 1.02–1.30; recessive: OR 1.58, 95% CI: 1.32–1.89). The CT genotype of miR‐149 (rs2292832) was related to lower CRC risk than TT genotype (OR 0.82, 95% CI: 0.68–0.99). The recessive (OR 0.73, 95% CI: 0.60–0.88) and over‐dominant models (OR 0.86, 95% CI: 0.75–0.99) of miR‐608 (rs4919510) showed protective roles against CRC. The AA genotype of miR‐143/145 (rs41291957) conferred a lower CRC risk than AG and GG genotypes (OR 0.76, 95% CI: 0.58–0.99). We observed dual effects of miR‐143/145 (rs353292) on CRC susceptibility, with allelic (OR 2.17, 95% CI: 1.13–4.15), heterozygote (OR 56.44, 95% CI: 6.99–455.86), and dominant models (OR 2.46, 95% CI: 1.23–4.91) predisposing to CRC, but over‐dominant model (OR 0.40, 95% CI: 0.21–0.76) protecting against CRC. All genetic models of miR‐143/145 (rs4705341), with the exception of heterozygote and over‐dominant models, correlated with reduced CRC risk (allelic: OR 0.77, 95% CI: 0.63–0.93; homozygote: OR 0.62, 95% CI: 0.48–0.79; dominant: OR 0.69, 95% CI: 0.49–0.98; recessive: OR 0.70, 95% CI: 0.56–0.89). The C allele and CC genotype of miR‐34b/c (rs4938723) conferred a lower CRC risk under the allele contrast (OR 0.82, 95% CI: 0.74–0.90), homozygote (OR 0.63, 95% CI: 0.51–0.79), and recessive models (OR 0.69, 95% CI: 0.56–0.85), respectively. The other miRNA‐SNPs did not show significant associations with CRC susceptibility. Data S4 summarized the detailed results of pairwise meta‐analyses.

### 3.3 Heterogeneity and Publication Bias

Subgroup/meta regression analysis and a leave‐one‐out method were employed to investigate sources of heterogeneity. For all leave‐one‐out analyses, the between‐study heterogeneities persisted, suggesting that the results were reliable. The Egger's and Bgger's test indicated that no obvious bias of publication was detected among studies (Data S4).

### 3.4 Candidate Genetic Models

**FIGURE 2:**
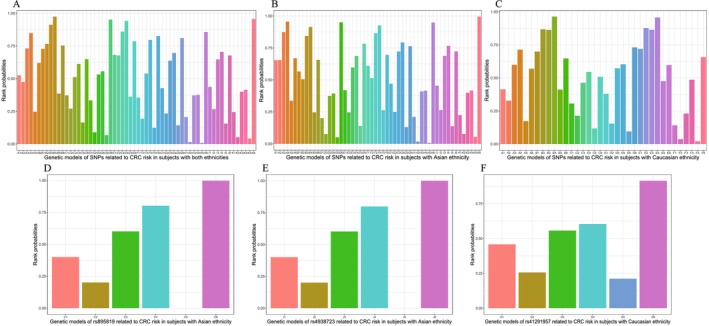
Rank probabilities of genetic models of the miRNA‐SNPs susceptible to CRC of Caucasian ethnicity (C, F), Asian ethnicity (B, D, and E), and both ethnicities (A). miRNA‐SNPs: (A) miR‐196a2 (rs11614913), (B) miR‐146a (rs2910164), (C) miR‐27a (rs895819), (D) miR‐499 (rs3746444), (E) miR‐149 (rs2292832), (F) miR‐608 (rs4919510), (G) miR‐143/145 (rs41291957), (H) miR‐143/145 (rs353292), (I) miR‐143/145 (rs4705341), (J) miR‐34b/c (rs4938723), (K) miR‐124 (rs531564). Genetic models: (1) allele contrast model, (2) homozygous model, (3) heterozygous model, (4) dominant model, (5) recessive model, and (6) over‐dominant model. *Note:* The genetic models with larger SUCRA scores are considered to be more suitable models for predicting CRC risk.

The network meta‐analysis with consistency model was conducted to rank the models of all the miRNA‐SNPs for predicting CRC risk. The findings indicated that all SNPs were connected to single mesh, except for miR‐34b/c (rs4938723) that was connected solely with its own genetic models (Data S5). All genetic models of the SNPs demonstrated convergence, with the PSRF value approaching 1.0. The rank probability indicated that the most suitable genetic models were either dominant or over‐dominant (Figure 2A–F, Table 3). The Thakkinstian's algorithm results showed that either dominant or recessive models were the most suitable (Table 3). Two distinctive genetic models of each miRNA‐SNP might be respectively proposed by two methods as the most suitable. The genetic models significantly correlated with CRC risk in pairwise meta‐analyses would be regarded as the candidate genetic models. Finally, in the present study, 10 candidate genetic models were identified for 8 miRNA‐SNPs, which might predispose to or protect against CRC among specific ethnicities (Table 3).

**TABLE 3:** Screening optimal genetic models of miRNA‐SNPs for predicting CRC risk.

miRNA‐SNPs^a^	No. of studies^b^	Ethnicity	Relevant genetic models	Network meta‐analysis evaluation^c^	Thakkinstian's algorithm results^c^	Candidate genetic models	FPRP evaluation^d^	Definitive genetic models	Predisposition to CRC
miR‐196a2 (rs11614913)	14	Both	Dominant	Dominant	NA	Dominant	0.784	/	
miR‐196a2 (rs11614913)	6	Asian	Allele, homozygote, heterozygote, dominant and recessive	Dominant	Dominant	Dominant	0.004	Dominant	Protective
miR‐27a (rs895819)	7	Both	Allele, homozygote, dominant and recessive	Over‐dominant	Recessive	Recessive	< 0.001	Recessive	Hazardous
miR‐27a (rs895819)	4	Asian	Homozygote and recessive	Over‐dominant	Recessive	Recessive	0.005	Recessive	Hazardous
miR‐27a (rs895819)	3	Caucasian	Allele, homozygote and recessive	Dominant	NA	/	/	/	
miR‐149 (rs2292832)	4	Both	Heterozygote	Dominant	NA	/	/	/	
miR‐149 (rs2292832)	2	Caucasian	Allele, homozygote and dominant	Dominant	NA	Dominant	0.939	/	
miR‐608 (rs4919510)	3	Both	Recessive and over‐dominant	Over‐dominant	NA	Over‐dominant	0.780	/	
miR‐143/145 (rs41291957)	5	Both	Recessive	Over‐dominant	NA	/	/	/	
miR‐143/145 (rs41291957)	4	Asian	Homozygote and recessive	Dominant	Recessive	Recessive	0.013	Recessive	Protective
miR‐143/145 (rs353292)	2	Asian	Allele, heterozygote, dominant and over‐dominant	Over‐dominant	NA	Over‐dominant	0.896	/	
miR‐143/145 (rs4705341)	2	Asian	Allele, homozygote, dominant, recessive	Dominant	NA	Dominant	0.868	/	
miR‐34b/c (rs4938723)	3	Asian	Allele, homozygote and recessive	Over‐dominant	Recessive	Recessive	0.072	Recessive	Protective

Abbreviation: FPRP, false positive report probability.

^a^ miRNA‐SNPs with significant results in pairwise meta‐analysis.

^b^ A minimum of two studies were needed for the assessment of optimal models.

^c^ Optimal genetic models were determined by network meta‐analysis or Thakkinstian's Algorithm or both.

^d^ Noteworthy results by FPRP analysis (by setting an OR of 1.5, FPRP value < 0.2 under the prior probabilities of below 0.01) were obtained.

### 3.5 Definitive Genetic Models

**FIGURE 3:**
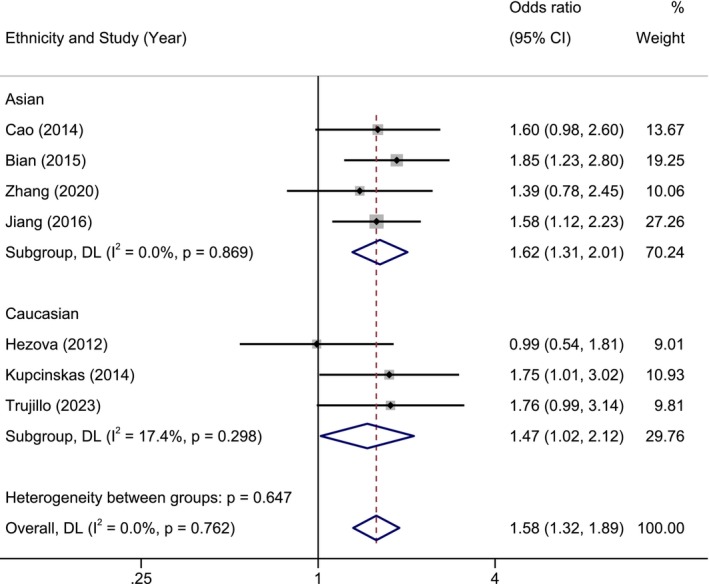
Subgroup analysis of the recessive genetic model of miRNA‐27a (rs895819) in Caucasian and Asian groups. Weights and between‐subgroup heterogeneity test are from random‐effects model.

The Excel spreadsheet for FPRP calculations was applied to determine whether the candidate genetic models were noteworthy [30]. The FPRPs were computed under five prior probabilities by setting an OR of 1.5. According to our predetermined criteria, the candidate genetic models with the FPRP less than 0.2, under the prior probability of 0.01, would be regarded as the definitive genetic models. In the present study, 5 definitive genetic models were determined for 4 miRNA‐SNPs, including the dominant model of miR‐196a2 (rs11614913) in ethnicity of Asian, recessive models of miR‐27a (rs895819) among Asians or overall population, recessive model of miR‐143/145 (rs41291957) among Asians, and recessive model of miR‐34b/c (rs4938723) among individuals from Asia (Table 3). Of the 4 miRNA‐SNPs, miR‐27a (rs895819) was deleterious and could considerably predispose the overall population or Asians to CRC, while the remaining miRNA‐SNPs were found to be protective exclusively among Asians (Table 3). The forest plot showed the results of pairwise meta‐analysis with ethnicity‐based subgroup analysis for miR‐27a (rs895819) (Figure 3).

### 3.6 Diagnostic Performance of the Optimal Genetic Models Predisposing to CRC

**FIGURE 4:**
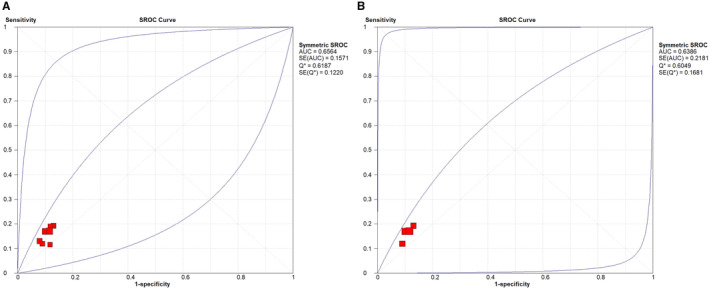
Summary receiver‐operating characteristic (SROC) curve of miR‐27a (rs895819) for identifying CRC under the recessive model among overall population (A), and Asian population (B).

The diagnostic meta‐analysis was conducted to evaluate the role of miR‐27a (rs895819) in diagnosing CRC. Among overall population and Asians, the Spearman correlation coefficients under recessive genetic model were 0.607 (*p* = 0.148) and 1.00 (*p* < 0.001), respectively. Random effects models were used, as the heterogeneity may be attributed to the threshold effect. Among overall population, the AUC was 0.656 (SE, 0.1571). The pooled DOR was 1.58 (95% CI, 1.32–1.89). The pooled sensitivity and specificity were 0.16 (95% CI, 0.14–0.18) and 0.90 (95% CI, 0.88–0.91), respectively. For Asians, the AUC was 0.639 (SE, 0.2181). The pooled DOR was 1.62 (95% CI, 1.31–2.01). The summary sensitivity and specificity were 0.17 (95% CI, 0.15–0.19) and 0.89 (95% CI, 0.88–0.91), respectively. Figure 4 presented the SROC curves of rs895819 for identifying CRC under the recessive model among overall population and Asians.

## 4 Discussion

From the combined evaluation by meta‐analysis, the Thakkinstian's method and the FPRP criterion, the present study demonstrated that rs895819 was significantly related to enhanced CRC risk among overall population (OR 1.58, 95% CI: 1.32–1.89) and Asians (OR 1.62, 95% CI: 1.31–2.01), with the recessive model identified as the most optimal. Nonetheless, rs11614913, rs41291957, and rs4938723 all exhibited protective effects against CRC among individuals from Asia under the optimal dominant (OR 0.75, 95% CI: 0.65–0.86), recessive (OR 0.72, 95% CI: 0.60–0.85), and recessive models (OR 0.69, 95% CI: 0.56–0.85), respectively. In the present study, we observed that the miRNA‐SNPs displayed a relatively stronger association with CRC risk in Asians compared to Caucasians, potentially due to the discrepancies in genetic settings and the lifestyle/environmental factors.

In the last decade, considerable systematic reviews and meta‐analyses regarding numerous miRNA‐SNPs in association with CRC susceptibility have been conducted. Nevertheless, no consistent results have been achieved. In addition, only pairwise meta‐analysis was used in previous studies; hence the most appropriate miRNAs with noteworthy genetic models for predicting CRC risk could not be determined. Moreover, most of the studies looked into a single or a limited number of miRNA‐SNPs, making it impossible to comprehensively figure out how miRNA‐SNPs affect the risk of CRC. Herein, an overview of previous published meta‐analyses was made chronologically as follows. As for rs895819, in Ma et al.'s study, they failed to found any correlation between rs895819 polymorphisms and CRC risk in the overall population and in subgroup analysis [71]. Of note, in this meta‐analysis, only one study indicating no correlation of rs895819 with CRC risk in Caucasians was included [53], hence a subgroup analysis to concentrate on rs895819's role in CRC carcinogenesis was not possible. Liu and colleagues have found that the rs895819 polymorphism could predispose the Chinese population to CRC under allelic, homozygote, dominant and recessive models [72]. A study conducted by Pan and colleagues included only three studies, and showed that rs895819 increased CRC risk under homozygous, dominant and allelic models [73]. In a meta‐analysis with seven trials included, Yang et al. [74] reported that the rs895819 was related to enhanced CRC susceptibility under recessive, homozygous, and allelic models. Dai and colleagues [75] have conducted an updated meta‐analysis yielding similar results. In contrast, Rong et al. [76] found no association of rs895819 with CRC susceptibility most likely because only two studies were identified to pool results.

As for rs11614913 in miR‐196a2, most prior meta‐analyses supported its protective role against CRC under distinct genetic models, especially among individuals from Asia [71, 77, 78, 79, 80, 81, 82, 83]. Among these studies, three reported that rs11614913 could predispose individuals to CRC while the C/T polymorphisms were evaluated, contrasting to the T/C polymorphism in the present study [81, 82, 83]. There were also several studies which found no association between the rs11614913 and CRC risk [73, 76, 84]. Regarding the SNP rs41291957 in miRNA‐143/145, Harati‐Sadegh and colleagues [85] demonstrated that this polymorphism showed protective effects against CRC. Feng et al. [70] found that the rs41291957 polymorphism might be a plausible locus for CRC risk. However, in this study, the G/A polymorphism, as opposed to A/G from our study, was evaluated, thus their findings were deemed consistent with the present study. As regards to the rs4938723 variant of miRNA‐34b/c, the relationship between this SNP and cancer risk in digestive system, including CRC, was not found in a study conducted by Wang and colleagues [86]. In another meta‐analysis with two studies included, Hashemi et al. demonstrated that the rs4938723 was related to reduced CRC risk under homozygous and recessive models [87].

In the present study, several miRNA‐SNPs were identified as relevant solely by pairwise meta‐analysis. However, they could not be assessed as the most appropriate or noteworthy polymorphisms in relation to CRC susceptibility. In pairwise meta‐analysis, we observed that miR‐149 (rs2292832) could protect individuals from CRC risk, especially among Caucasians. Some meta‐analyses failed to find any associations of the rs2292832 polymorphism with CRC risk [71, 83], while this polymorphism was found to correlate with reduced CRC risk in some other studies [73, 76, 79, 80, 84]. In a case–control study, the allele G and genotype GG of rs4919510 in miRNA‐608 have been identified as protective factors for stage 0‐II CRC among Asians [61]. While in another two case–control studies involving Caucasian population, no association of this SNP with CRC risk was found [58, 67]. After pooling the results of these two studies, we still failed to find any significant results for this SNP in Caucasian population, which was also in agreement with a previously published study [88].

To date, there have been three comprehensively designed meta‐analyses from a field perspective regarding this topic. The initial two studies did not include any SNPs in miRNA genes, perhaps due to the early timeframe established for retrieval, during which no relevant studies had been published [26, 28]. In 2018, Wen et al. [27] performed an updated synopsis involving SNPs' susceptibility to sporadic CRC. Using the Venice criteria, they discovered 15 high‐quality polymorphisms, among which miR‐196a2 (rs11614913) was found to reduce CRC risk, while miR‐27a (rs895819) increased CRC risk, particularly in Asian population [27].

The findings of this study indicate that those with the rs895819 GG genotype may be related to CRC susceptibility. The other miRNA‐SNPs with definitive genetic models were found to be protective against CRC solely among population from Asia (Table 2). Furthermore, the rs895819 polymorphism could be the most appropriate miRNA‐SNP associated with CRC predisposition, with an FPRP value of 0.164 at a highly stringent prior probability of 0.00001. miRNA‐27a is mapped on chromosome 19 (19p13.13) and related to the development of multiple cancers. The corresponding SNP (rs895819) is situated within the loop of pre‐miR‐27, resulting in the nucleotide A>G alteration in this locus. In colon cancer, miR‐27a may have an effect on promoting lymphangiogenesis and migration by targeting SMAD4 [89]. In gastric cancer, the rs895819 has been found to affect the expression of pri‐miRNA‐27a or pre‐miRNA‐27a [90]. Moreover, the rs895819 variant was an important factor of gastric cancer predisposition by regulating miRNA‐27a and ZBTB10 levels [91]. Based on these findings, we hypothesize that these mechanisms may also influence CRC susceptibility.

An increasing number of studies have revealed that microRNAs can post‐transcriptionally regulate the expression of about 10%–30% of human genes [92], including oncogenes and tumor suppressor genes, which are strongly related to the development, prognosis, and treatment of cancer. The well‐known Vogelstein model of the adenoma‐carcinoma sequence described the progression of cancer as a multistep model characterized by the mutational activation/inactivation of a series of oncogenes and/or tumor suppressors [93]. In fact, the tumorigenesis of sporadic CRC has been observed to involve hereditary factors involving sequential mutations of several critical oncogenes/tumor suppressor genes, as well as numerous environmental factors, including aging, socioeconomic status, physical activity, dietary habits, and smoking, etc. Each individual miRNA‐SNP may be related to only a little increase/decrease in cancer susceptibility, which can be discerned in the results of our diagnostic meta‐analyses. Each individual miRNA‐SNP may exhibit interactions with other SNPs and environmental factors. Carriers of several miRNA‐SNPs, as promising biomarkers, may confer greater susceptibility to CRC.

Genetic association analysis utilizing a pre‐established genetic model may produce misleading results. Herein a completely unbiased analysis was conducted, and thus the relationship between miRNA‐SNPs and CRC susceptibility was assessed under six genetic models. As far as we know, we are the first to investigate the association between the SNPs and CRC risk using pairwise and network meta‐analysis, as well as the Thakkinstian's algorithm and the FPRP criterion. Nevertheless, there are still several shortcomings in our study. Firstly, only publications involving Asian and Caucasian populations were included in our study, hence our findings might only be extrapolated to these ethnic groups. Secondly, the pooled results showed significant heterogeneity among studies that subgroup analysis and meta‐regression failed to fully explain. This may suggest the underlying impact of unexamined confounders in the present study. Thirdly, the potential influences of many extrinsic factors including gender, age, smoking, drinking and nutrient intake among others, which were largely unavailable in original studies, were not considered. Lastly, given the small sample sizes in the analyses for several polymorphisms, caution should be taken in interpreting the results.

## 5 Conclusion

The present study provides evidence supporting miR‐196a2 (rs11614913), miR‐143/145 (rs41291957), and miR‐34b/c (rs4938723) as protective miRNA‐SNPs against CRC among Asians, with dominant, recessive, and recessive models being the optimal, respectively. Contrarily, the miR‐27a (rs895819) polymorphism appears to be related to enhanced CRC risk among overall population and Asians, and the recessive model was found to be optimal for predicting CRC risk. Several miRNA‐SNPs, assessed as relevant SNPs solely by pairwise meta‐analysis, were not identified as the definitive SNPs with noteworthy genetic models for predicting CRC risk, perhaps due to the restricted quantity of trials included. More high‐quality trials with large samples, detailed information concerning environmental factors, and involving multiple ethnicities are needed to validate our findings and further investigate the role of gene–gene/gene–environment interactions in the development of CRC.

## Author Contributions

**Qing Liu:** data curation (equal); formal analysis (equal); investigation (equal); methodology (equal); software (equal); writing – original draft (equal). **Ivan Archilla:** investigation (equal); data curation (equal); methodology (equal); writing – review and editing (equal). **Sandra Lopez‐Prades:** investigation (equal); writing – review and editing (equal). **Ferran Torres:** methodology (equal); software (equal); writing – review and editing (equal). **Jordi Camps:** conceptualization (equal); supervision (equal); writing – review and editing (equal). **Miriam Cuatrecasas:** conceptualization (equal); supervision (equal); writing – review and editing (equal).

## Conflicts of Interest

The authors declare no conflicts of interest.

## Supporting information


Data S1–S2.

Data S3.

Data S4.

Data S5.

## Data Availability

The data in the present study are derived from previously published studies, as shown in the references list. The processed data can be obtained from the corresponding authors upon request.

## References

[1] F. Bray , M. Laversanne , H. Sung , et al., “Global Cancer Statistics 2022: GLOBOCAN Estimates of Incidence and Mortality Worldwide for 36 Cancers in 185 Countries,” CA: A Cancer Journal for Clinicians 74 (2024): 229–263. 38572751 10.3322/caac.21834

[2] J. Bogaert and H. Prenen , “Molecular Genetics of Colorectal Cancer,” Annals of Gastroenterology 27 (2014): 9–14. 24714764 PMC3959535

[3] F. Rebuzzi , P. Ulivi , and G. Tedaldi , “Genetic Predisposition to Colorectal Cancer: How Many and Which Genes to Test?,” International Journal of Molecular Sciences 24 (2023): 2137. 36768460 10.3390/ijms24032137 PMC9916931

[4] A. A. Abd El‐Fattah , N. A. H. Sadik , O. G. Shaker , et al., “Single Nucleotide Polymorphism in SMAD7 and CHI3L1 and Colorectal Cancer Risk,” Mediators of Inflammation 2018 (2018): 9853192. 30498395 10.1155/2018/9853192 PMC6222239

[5] A. de la Chapelle , “Genetic Predisposition to Colorectal Cancer,” Nature Reviews. Cancer 4 (2004): 769–780. 15510158 10.1038/nrc1453

[6] S. Y. Jung , J. C. Papp , E. M. Sobel , and Z. F. Zhang , “Post Genome‐Wide Gene‐Environment Interaction Study Using Random Survival Forest: Insulin Resistance, Lifestyle Factors, and Colorectal Cancer Risk,” Cancer Prevention Research 12 (2019): 877–890. 31554631 10.1158/1940-6207.CAPR-19-0278 PMC6893139

[7] N. Dimou , A. E. Kim , O. Flanagan , et al., “Probing the Diabetes and Colorectal Cancer Relationship Using Gene—Environment Interaction Analyses,” British Journal of Cancer 129 (2023): 511–520. 37365285 10.1038/s41416-023-02312-z PMC10403521

[8] M. C. Stern , J. Sanchez Mendez , A. E. Kim , et al., “Genome‐Wide Gene‐Environment Interaction Analyses to Understand the Relationship Between Red Meat and Processed Meat Intake and Colorectal Cancer Risk,” Cancer Epidemiology, Biomarkers & Prevention 33 (2024): 400–410. 10.1158/1055-9965.EPI-23-0717 PMC11343583 38112776

[9] T. Yang , X. Li , Z. Montazeri , et al., “Gene–Environment Interactions and Colorectal Cancer Risk: An Umbrella Review of Systematic Reviews and Meta‐Analyses of Observational Studies,” International Journal of Cancer 145 (2019): 2315–2329. 30536881 10.1002/ijc.32057 PMC6767750

[10] X. Zhou , Q. Xiao , F. Jiang , et al., “Dissecting the Pathogenic Effects of Smoking and Its Hallmarks in Blood DNA Methylation on Colorectal Cancer Risk,” British Journal of Cancer 129 (2023): 1306–1313. 37608097 10.1038/s41416-023-02397-6 PMC10576058

[11] C. M. Hutter , J. Chang‐Claude , M. L. Slattery , et al., “Characterization of Gene‐Environment Interactions for Colorectal Cancer Susceptibility Loci,” Cancer Research 72 (2012): 2036–2044. 22367214 10.1158/0008-5472.CAN-11-4067 PMC3374720

[12] P. Losurdo , M. Giacca , A. Biloslavo , et al., “Colorectal Cancer‐Screening Program Improves Both Short‐ and Long‐Term Outcomes: A Single‐Center Experience in Trieste,” Updates in Surgery 72 (2020): 89–96. 31965546 10.1007/s13304-020-00703-y

[13] J. Cubiella , M. Lorenzo , F. Baiocchi , et al., “Impact of a Colorectal Cancer Screening Program Implantation on Delays and Prognosis of Non‐Screening Detected Colorectal Cancer,” World Journal of Gastroenterology 27 (2021): 6689–6700. 34754161 10.3748/wjg.v27.i39.6689 PMC8554396

[14] J. S. Hunt , C. Cock , and E. L. Symonds , “A True Positive and a False Negative? The Dilemma of Negative Colonoscopy After a Positive Fecal Occult Blood Test,” Digestive Diseases and Sciences 67 (2022): 1843–1849. 33939150 10.1007/s10620-021-06986-4

[15] S. B. Therkildsen , P. T. Larsen , and S. H. Njor , “Screening Participants With Inflammatory Bowel Disease or High Colorectal Cancer Risk in Denmark: A Cohort Study,” Journal of Public Health Policy 45 (2024): 727–739. 39414911 10.1057/s41271-024-00523-z PMC11609084

[16] S. Lin and R. I. Gregory , “MicroRNA Biogenesis Pathways in Cancer,” Nature Reviews. Cancer 15 (2015): 321–333. 25998712 10.1038/nrc3932 PMC4859809

[17] Z. Ali Syeda , S. S. S. Langden , C. Munkhzul , et al., “Regulatory Mechanism of MicroRNA Expression in Cancer,” International Journal of Molecular Sciences 21 (2020): 1723. 32138313 10.3390/ijms21051723 PMC7084905

[18] Y. S. Lee and A. Dutta , “MicroRNAs in Cancer,” Annual Review of Pathology 4 (2009): 199–227. 10.1146/annurev.pathol.4.110807.092222 PMC2769253 18817506

[19] G. A. Calin , C. Sevignani , C. D. Dumitru , et al., “Human microRNA Genes Are Frequently Located at Fragile Sites and Genomic Regions Involved in Cancers,” National Academy of Sciences of the United States of America Proceedings of the National Academy of Sciences 101 (2004): 2999–3004. 10.1073/pnas.0307323101 PMC365734 14973191

[20] F. S. Collins , L. D. Brooks , and A. Chakravarti , “A DNA Polymorphism Discovery Resource for Research on Human Genetic Variation,” Genome Research 8 (1998): 1229–1231. 9872978 10.1101/gr.8.12.1229

[21] B. M. Ryan , A. I. Robles , and C. C. Harris , “Genetic Variation in microRNA Networks: The Implications for Cancer Research,” Nature Reviews. Cancer 10 (2010): 389–402. 20495573 10.1038/nrc2867 PMC2950312

[22] M. Budakoti , A. S. Panwar , D. Molpa , et al., “Micro‐RNA: The Darkhorse of Cancer,” Cellular Signalling 83 (2021): 109995. 33785398 10.1016/j.cellsig.2021.109995

[23] A. Ottaiano , M. Santorsola , M. Ianniello , et al., “Predictive Significance of FGFR4 p.G388R Polymorphism in Metastatic Colorectal Cancer Patients Receiving Trifluridine/Tipiracil (TAS‐102) Treatment,” Journal of Translational Medicine 22 (2024): 379. 38650006 10.1186/s12967-024-05184-w PMC11036552

[24] G. Polcaro , L. Liguori , V. Manzo , et al., “rs822336 Binding to C/EBPβ and NFIC Modulates Induction of PD‐L1 Expression and Predicts Anti‐PD‐1/PD‐L1 Therapy in Advanced NSCLC,” Molecular Cancer 23 (2024): 63. 38528526 10.1186/s12943-024-01976-2 PMC10962156

[25] F. J. Nassar , Z. S. Msheik , M. M. Itani , et al., “Circulating miRNA as Biomarkers for Colorectal Cancer Diagnosis and Liver Metastasis,” Diagnostics 11 (2021): 341. 33669508 10.3390/diagnostics11020341 PMC7921943

[26] X. Ma , B. Zhang , and W. Zheng , “Genetic Variants Associated With Colorectal Cancer Risk: Comprehensive Research Synopsis, Meta‐Analysis, and Epidemiological Evidence,” Gut 63 (2014): 326–336. 23946381 10.1136/gutjnl-2012-304121 PMC4020522

[27] J. Wen , Q. Xu , and Y. Yuan , “Single Nucleotide Polymorphisms and Sporadic Colorectal Cancer Susceptibility: A Field Synopsis and Meta‐Analysis,” Cancer Cell International 18 (2018): 155. 30337837 10.1186/s12935-018-0656-2 PMC6180373

[28] E. Theodoratou , Z. Montazeri , S. Hawken , et al., “Systematic Meta‐Analyses and Field Synopsis of Genetic Association Studies in Colorectal Cancer,” Journal of the National Cancer Institute 104 (2012): 1433–1457. 23019048 10.1093/jnci/djs369

[29] A. Thakkinstian , P. McElduff , C. D'Este , D. Duffy , and J. Attia , “A Method for Meta‐Analysis of Molecular Association Studies,” Statistics in Medicine 24 (2005): 1291–1306. 15568190 10.1002/sim.2010

[30] S. Wacholder , S. Chanock , M. Garcia‐Closas , et al., “Assessing the Probability That a Positive Report Is False: An Approach for Molecular Epidemiology Studies,” JNCI Journal of the National Cancer Institute 96 (2004): 434–442. 15026468 10.1093/jnci/djh075 PMC7713993

[31] J. Zamora , V. Abraira , A. Muriel , K. Khan , and A. Coomarasamy , “Meta‐DiSc: A Software for Meta‐Analysis of Test Accuracy Data,” BMC Medical Research Methodology 6 (2006): 31. 16836745 10.1186/1471-2288-6-31 PMC1552081

[32] L. Zhu , H. Chu , D. Gu , et al., “A Functional Polymorphism in *miRNA‐196a2* Is Associated With Colorectal Cancer Risk in a Chinese Population,” DNA and Cell Biology 31 (2012): 350–354. 21815818 10.1089/dna.2011.1348

[33] J. Zhan , L. Chen , Z. Chen , et al., “A Functional Variant in MicroRNA‐196a2 Is Associated With Susceptibility of Colorectal Cancer in a Chinese Population,” Archives of Medical Research 42 (2011): 144–148. 21565628 10.1016/j.arcmed.2011.04.001

[34] F. Yuan , R. Sun , L. Li , et al., “A Functional Variant rs353292 in the Flanking Region of miR‐143/145 Contributes to the Risk of Colorectal Cancer,” Scientific Reports 6 (2016): 30195. 27444415 10.1038/srep30195 PMC4957080

[35] L. Ma , L. Zhu , D. Gu , et al., “A Genetic Variant in miR‐146a Modifies Colorectal Cancer Susceptibility in a Chinese Population,” Archives of Toxicology 87 (2013): 825–833. 23306950 10.1007/s00204-012-1004-2

[36] L.‐B. Gao , L.‐J. Li , X.‐M. Pan , et al., “A Genetic Variant in the Promoter Region of miR‐34b/c Is Associated With a Reduced Risk of Colorectal Cancer,” Biological Chemistry 394 (2013): 415–420. 23183747 10.1515/hsz-2012-0297

[37] Y. S. Chae , J. G. Kim , S. J. Lee , et al., “A miR‐146a Polymorphism (rs2910164) Predicts Risk of and Survival From Colorectal Cancer,” Anticancer Research 33 (2013): 3233–3239. 23898084

[38] R. Sun , P. Chen , L. Li , et al., “A Polymorphism rs4705341 in the Flanking Region of miR‐143/145 Predicts Risk and Prognosis of Colorectal Cancer,” Oncotarget 7 (2016): 62084–62090. 27556691 10.18632/oncotarget.11387 PMC5308712

[39] H. Chen , L. Sun , L. Chen , et al., “A Variant in microRNA‐196a2 Is Not Associated With Susceptibility to and Progression of Colorectal Cancer in Chinese,” Internal Medicine Journal 42 (2012): e115–e119. 21241442 10.1111/j.1445-5994.2011.02434.x

[40] D. Wang , Q. Liu , Y. Ren , et al., “Association Analysis of miRNA‐Related Genetic Polymorphisms in miR‐143/145 and KRAS With Colorectal Cancer Susceptibility and Survival,” Bioscience Reports 41 (2021): BSR20204136. 33825830 10.1042/BSR20204136 PMC8062955

[41] C. Parlayan , S. Ikeda , N. Sato , M. Sawabe , M. Muramatsu , and T. Arai , “Association Analysis of Single Nucleotide Polymorphisms in miR‐146a and miR‐196a2 on the Prevalence of Cancer in Elderly Japanese: A Case‐Control Study,” Asian Pacific Journal of Cancer Prevention 15 (2014): 2101–2107. 24716941 10.7314/apjcp.2014.15.5.2101

[42] Y. Cao , J. Hu , Y. Fang , Q. Chen , and H. Li , “Association Between a Functional Variant in MicroRNA‐27a and Susceptibility to Colorectal Cancer in a Chinese Han Population,” Genetics and Molecular Research 13 (2014): 7420–7427. 25222241 10.4238/2014.September.12.8

[43] M. Lv , W. Dong , L. Li , et al., “Association Between Genetic Variants in Pre‐miRNA and Colorectal Cancer Risk in a Chinese Population,” Journal of Cancer Research and Clinical Oncology 139 (2013): 1405–1410. 23728616 10.1007/s00432-013-1456-7 PMC11824633

[44] X. Hu , L. Li , M. Shang , et al., “Association Between microRNA Genetic Variants and Susceptibility to Colorectal Cancer in Chinese Population,” Tumor Biology 35 (2014): 2151–2156. 24136745 10.1007/s13277-013-1285-y

[45] L. Li , X. Pan , Z. Li , et al., “Association Between Polymorphisms in the Promoter Region of miR‐143/145 and Risk of Colorectal Cancer,” Human Immunology 74 (2013): 993–997. 23628392 10.1016/j.humimm.2013.04.019

[46] J. Xu , Q. Bian , J.‐J. Chen , and J. P. Gu , “Association Between Pre‐miR‐27a Functional Polymorphism and Risk of Colorectal Cancer in&Nbsp;North Chinese Han Population,” OncoTargets and Therapy 8 (2015): 3003–3007. 26527885 10.2147/OTT.S89754 PMC4621200

[47] A. R. Soltanian and N. Mojarad , “Association Between rs11614913 Polymorphism of the MiR‐196‐a2 Gene and Colorectal Cancer in the Presence of Departure From Hardy–Weinberg Equilibrium,” Cell 23 (2021): 313–318. 10.22074/cellj.2021.7295 PMC8286463 34308574

[48] Y. Mao , Y. Li , F. Jing , et al., “Association of a Genetic Variant in microRNA‐146a With Risk of Colorectal Cancer: A Population‐Based Case–Control Study,” Tumor Biology 35 (2014): 6961–6967. 24740563 10.1007/s13277-014-1916-y

[49] K. T. Min , J. W. Kim , Y. J. Jeon , et al., “Association of the *miR‐146* aC>G, *149* C>T, *196a2* C>T, and *499* A>G Polymorphisms With Colorectal Cancer in the Korean Population,” Molecular Carcinogenesis 51 (2012): E65–E73. 22161766 10.1002/mc.21849

[50] Z. Mw , J. Mj , Y. Yx , et al., “Associations of Lifestyle‐Related Factors, Hsa‐miR‐149 and Hsa‐miR‐605 Gene Polymorphisms With Gastrointestinal Cancer Risk,” Molecular Carcinogenesis 51 (2012): E21–E31. 21976437 10.1002/mc.20863

[51] V. Chayeb , S. Mahjoub , H. Zitouni , et al., “Contribution of MicroRNA‐149, microRNA‐146a, and microRNA‐196a2 SNPs in Colorectal Cancer Risk and Clinicopathological Features in Tunisia,” Gene 666 (2018): 100–107. 29715515 10.1016/j.gene.2018.04.084

[52] P. Dikaiakos , M. Gazouli , S. Rizos , G. Zografos , and G. E. Theodoropoulos , “Evaluation of Genetic Variants in miRNAs in Patients With Colorectal Cancer,” Cancer Biomarkers 15 (2015): 157–162. 25519012 10.3233/CBM-140449 PMC12928517

[53] R. Hezova , “Evaluation of SNPs in miR‐196‐a2, miR‐27a and miR‐146a as Risk Factors of Colorectal Cancer,” World Journal of Gastroenterology 18 (2012): 2827. 22719192 10.3748/wjg.v18.i22.2827 PMC3374987

[54] Y. G. V. Trujillo‐Fernández , C. Yzabal‐Barbedillo , A. M. Saucedo‐Sarinaña , et al., “Functional Variants in MicroRNAs (rs895819, rs11614913 and rs2910164) are Associated With Susceptibility and Clinicopathological Features in Mexican Patients With Colorectal Cancer,” Archives of Iranian Medicine 26 (2023): 439–446. 38301106 10.34172/aim.2023.67 PMC10685737

[55] S. Vinci , S. Gelmini , I. Mancini , et al., “Genetic and Epigenetic Factors in Regulation of microRNA in Colorectal Cancers,” Methods 59 (2013): 138–146. 22989523 10.1016/j.ymeth.2012.09.002

[56] Y. Jiang , D. Lin , J. Xu , et al., “Genotype GG of rs895819 Functional Polymorphism Within miR‐27a Might Increase Genetic Susceptibility to Colorectal Cancer in Han Chinese Population,” Journal of Clinical Laboratory Analysis 30 (2016): 351–355. 26302683 10.1002/jcla.21862 PMC6806710

[57] T.‐C. Yueh , Y.‐C. Wang , Y.‐T. Chin , et al., “Impact of Mir196a‐2 Genotypes on Colorectal Cancer Risk in Taiwan,” International Journal of Molecular Sciences 24 (2023): 11613. 37511371 10.3390/ijms241411613 PMC10380589

[58] J. Kupcinskas , I. Bruzaite , S. Juzenas , et al., “Lack of Association Between miR‐27a, miR‐146a, miR‐196a‐2, miR‐492 and miR‐608 Gene Polymorphisms and Colorectal Cancer,” Scientific Reports 4 (2014): 5993. 25103961 10.1038/srep05993 PMC4125984

[59] J. Jiang , S. Zhang , W. Tang , et al., “Lack of Association Between miR‐146a rs2910164 C/G Locus and Colorectal Cancer: From a Case–Control Study to a Meta‐Analysis,” Bioscience Reports 41 (2021): BSR20191729. 33289522 10.1042/BSR20191729 PMC7785042

[60] X. Gao , Z. Zhu , and S. Zhang , “miR‐146a rs2910164 Polymorphism and the Risk of Colorectal Cancer in Chinese Population,” Journal of Cancer Research and Therapeutics 14 (2018): S97–S99. 29578157 10.4103/0973-1482.165864

[61] H.‐Q. Ying , H.‐X. Peng , B.‐S. He , et al., “MiR‐608, Pre‐miR‐124‐1 and Pre‐miR26a‐1 Polymorphisms Modify Susceptibility and Recurrence‐Free Survival in Surgically Resected CRC Individuals,” Oncotarget 7 (2016): 75865–75873. 27713147 10.18632/oncotarget.12422 PMC5342784

[62] M. S. Haerian , B. S. Haerian , S. Molanaei , et al., “MIR196A2 rs11614913 Contributes to Susceptibility to Colorectal Cancer in Iranian Population: A Multi‐Center Case‐Control Study and Meta‐Analysis,” Gene 669 (2018): 82–90. 29802998 10.1016/j.gene.2018.05.082

[63] J. Oh , J. W. Kim , B. E. Lee , et al., “Polymorphisms of the Pri‐miR‐34b/c Promoter and TP53 Codon 72 Are Associated With Risk of Colorectal Cancer,” Oncology Reports 31 (2014): 995–1002. 24337371 10.3892/or.2013.2926

[64] M. N. Ahmed , Q. Mansoor , R. M. Baig , T. Tahir , and S. A. Abbasi , “Potential Risk Assessment of miR‐143 Gene Polymorphisms rs41291957 and rs353292 in Colorectal Cancer,” Cellular and Molecular Biology 66 (2020): 169–173. 33287937

[65] S. A. Kassim , X. Yang , M. Abbas , et al., “Pri‐miR‐34b/c rs4938723 Polymorphism Is Associated With Decreased Risk and Better Prognosis for Colorectal Cancer Patients,” Archives of Medical Research 50 (2019): 55–62. 31349954 10.1016/j.arcmed.2019.05.008

[66] X. Gao , H. Wang , S. Zhang , et al., “Pri‐miR‐124 rs531564 Polymorphism and Colorectal Cancer Risk,” Scientific Reports 5 (2015): 14818. 26423518 10.1038/srep14818 PMC4589757

[67] B. M. Ryan , A. C. McClary , N. Valeri , et al., “rs4919510 in hsa‐mir‐608 Is Associated with Outcome but Not Risk of Colorectal Cancer,” PLoS One 7 (2012): e36306. 22606253 10.1371/journal.pone.0036306 PMC3350523

[68] J. S. D. Santos , G. L. Zunta , A. B. Negrini , et al., “The Association of a Single‐Nucleotide Variant in the microRNA‐146a With Advanced Colorectal Cancer Prognosis,” Tumor Biology 42 (2020): 101042832092385. 10.1177/1010428320923856 32438863

[69] S. Zhang , Q. Han , K. Zhu , and Q. Wang , “The Association of miR‐27a rs895819 Polymorphism With Colorectal Cancer Risk in Chinese Population,” Journal of Clinical Laboratory Analysis 34 (2020): e23497. 32710451 10.1002/jcla.23497 PMC7676186

[70] L. Feng , Y. Jin , L. Chai , et al., “The rs41291957 Polymorphism of miR‐143/145 and Cancer Risk: A Case‐Control Study and Meta‐Analysis,” Nucleosides, Nucleotides & Nucleic Acids 42 (2023): 478–494. 10.1080/15257770.2022.2157436 36533675

[71] X. P. Ma , T. Zhang , B. Peng , et al., “Association Between microRNA Polymorphisms and Cancer Risk Based on the Findings of 66 Case–Control Studies,” PLoS One 8 (2013): e79584. 24278149 10.1371/journal.pone.0079584 PMC3835861

[72] F. Liu , K. Dear , L. Huang , et al., “Association Between MicroRNA‐27a rs895819 Polymorphism and Risk of Colorectal Cancer: A Meta‐Analysis,” Cancer Genetics 209 (2016): 388–394. 27751356 10.1016/j.cancergen.2016.08.003

[73] X. M. Pan , X. Xiao , H. J. Qin , et al., “MicroRNA Variants and Colorectal Cancer Risk: A Meta‐Analysis,” Genetics and Molecular Research 15 (2016): gmr.15038478. 10.4238/gmr.15038478 27706637

[74] X. Yang , X. Li , X. Hao , W. Tian , and B. Zhou , “Association of miR‐27a Polymorphism With the Risk of Digestive System Cancers,” Pathology, Research and Practice 216 (2020): 153115. 32853952 10.1016/j.prp.2020.153115

[75] J. Dai , Y. Chen , Y. Gong , D. Gu , and J. Chen , “Association of MicroRNA‐27a rs895819 Polymorphism With the Risk of Cancer: An Updated Meta‐Analysis,” Gene 728 (2020): 144185. 31838252 10.1016/j.gene.2019.144185

[76] G.‐Q. Rong , X.‐M. Zhang , B. Chen , X. D. Yang , H. R. Wu , and W. Gong , “MicroRNA Gene Polymorphisms and the Risk of Colorectal Cancer,” Oncology Letters 13 (2017): 3617–3623. 28521462 10.3892/ol.2017.5885 PMC5431414

[77] B. He , Y. Pan , W. C. Cho , et al., “The Association Between Four Genetic Variants in MicroRNAs (rs11614913, rs2910164, rs3746444, rs2292832) and Cancer Risk: Evidence From Published Studies,” PLoS One 7 (2012): e49032. 23155448 10.1371/journal.pone.0049032 PMC3498348

[78] K. Srivastava and A. Srivastava , “Comprehensive Review of Genetic Association Studies and Meta‐Analyses on miRNA Polymorphisms and Cancer Risk,” PLoS One 7 (2012): e50966. 23226435 10.1371/journal.pone.0050966 PMC3511416

[79] W. Du , X.‐L. Ma , C. Zhao , et al., “Associations of Single Nucleotide Polymorphisms in miR‐146a, miR‐196a, miR‐149 and miR‐499 With Colorectal Cancer Susceptibility,” Asian Pacific Journal of Cancer Prevention 15 (2014): 1047–1055. 24568449 10.7314/apjcp.2014.15.2.1047

[80] X.‐X. Liu , M. Wang , D. Xu , et al., “Quantitative Assessment of the Association Between Genetic Variants in MicroRNAs and Colorectal Cancer Risk,” BioMed Research International 2015 (2015): 1–10. 10.1155/2015/276410 PMC4452836 26078942

[81] K.‐Q. Shi , Z. Lin , D.‐W. Li , et al., “Meta‐Analysis of the Association Between a Polymorphism in MicroRNA‐196a2 and Susceptibility to Colorectal Cancer,” Oncology Research and Treatment 36 (2013): 560–565. 10.1159/000355158 24107909

[82] N. Wang , Y. Li , L.‐J. Zhu , et al., “A Functional Polymorphism rs11614913 in microRNA‐196a2 Is Associated With an Increased Risk of Colorectal Cancer Although Not With Tumor Stage and Grade,” Biomedical Reports 1 (2013): 737–742. 24649020 10.3892/br.2013.146 PMC3917088

[83] L. Xu and W. Tang , “Associations of Polymorphisms in Mir‐196a2, Mir‐146a and Mir‐149 With Colorectal Cancer Risk: A Meta‐Analysis,” Pathology Oncology Research 22 (2016): 261–267. 26208586 10.1007/s12253-014-9843-1

[84] J. Choupani , Z. Nariman‐Saleh‐Fam , Z. Saadatian , E. Ouladsahebmadarek , A. Masotti , and M. Bastami , “Association of Mir‐196a‐2 rs11614913 and Mir‐149 rs2292832 Polymorphisms With Risk of Cancer: An Updated Meta‐Analysis,” Frontiers in Genetics 10 (2019): 186. 30930933 10.3389/fgene.2019.00186 PMC6429108

[85] M. Harati‐Sadegh , S. Sargazi , M. Saravani , R. Sheervalilou , S. Mirinejad , and R. Saravani , “Relationship Between *miR‐143/145* Cluster Variations and Cancer Risk: Proof From a Meta‐Analysis,” Nucleosides, Nucleotides & Nucleic Acids 40 (2021): 578–591. 10.1080/15257770.2021.1916030 33980135

[86] B. Shen , “Association Between miR34b/c Polymorphism rs4938723 and Cancer Risk: A Meta‐Analysis of 11 Studies Including 6169 Cases and 6337 Controls,” Medical Science Monitor 20 (2014): 1977–1982. 25326793 10.12659/MSM.892350 PMC4213004

[87] M. Hashemi , A. Moazeni‐Roodi , G. Bahari , M. Taheri , and S. Ghavami , “Association Between miR‐34b/c rs4938723 Polymorphism and Risk of Cancer: An Updated Meta‐Analysis of 27 Case–Control Studies,” Journal of Cellular Biochemistry 120 (2019): 3306–3314. 30203457 10.1002/jcb.27598

[88] Z.‐M. Dai , J.‐R. Lv , K. Liu , et al., “The Role of microRNA‐608 Polymorphism on the Susceptibility and Survival of Cancer: A Meta‐Analysis,” Aging 10 (2018): 1402–1414. 29909406 10.18632/aging.101476 PMC6046227

[89] Q. Xu , J.‐L. Tong , C.‐P. Zhang , Q. Xiao , X. L. Lin , and X. Y. Xiao , “miR‐27a Induced by Colon Cancer Cells in HLECs Promotes Lymphangiogenesis by Targeting SMAD4,” PLoS One 12 (2017): e0186718. 29065177 10.1371/journal.pone.0186718 PMC5655427

[90] B. Song , G. Yan , H. Hao , et al., “rs11671784 G/A and rs895819 A/G Polymorphisms Inversely Affect Gastric Cancer Susceptibility and miR‐27a Expression in a Chinese Population,” Medical Science Monitor 20 (2014): 2318. 25399405 10.12659/MSM.892499 PMC4245106

[91] Q. Sun , H. Gu , Y. Zeng , et al., “Hsa‐Mir‐27a Genetic Variant Contributes to Gastric Cancer Susceptibility Through Affecting miR‐27a and Target Gene Expression,” Cancer Science 101 (2010): 2241–2247. 20666778 10.1111/j.1349-7006.2010.01667.x PMC11159034

[92] E. Berezikov , V. Guryev , J. van de Belt , E. Wienholds , R. H. A. Plasterk , and E. Cuppen , “Phylogenetic Shadowing and Computational Identification of Human microRNA Genes,” Cell 120 (2005): 21–24. 15652478 10.1016/j.cell.2004.12.031

[93] B. Vogelstein , E. R. Fearon , S. R. Hamilton , et al., “Genetic Alterations During Colorectal‐Tumor Development,” New England Journal of Medicine 319 (1988): 525–532. 2841597 10.1056/NEJM198809013190901

