# Supplementary material for: Polymorphic Single‐Nucleotide Variants in miRNA Genes and the Susceptibility to Colorectal Cancer: Combined Evaluation by Pairwise and Network Meta‐Analysis, Thakkinstian's Algorithm and FPRP Criterium
Source: Cancer Med. 2025 Jan 22;14(2):e70621. doi: 10.1002/cam4.70621 (PMC11751872; doi:10.1002/cam4.70621)
Supplement: Supplementary file 1 — Data S1–S2. [file CAM4-14-e70621-s003.docx]

**Supplementary materials 1** Search strategy of studies

1. **Pubmed**

(("Colorectal Neoplasms"[Mesh]) OR ((((((((Colorectal Neoplasm*[Title/Abstract]) OR (Neoplasm*, Colorectal[Title/Abstract])) OR (Colorectal Tumor*[Title/Abstract])) OR (Tumor*, Colorectal[Title/Abstract])) OR (Colorectal Cancer*[Title/Abstract])) OR (Cancer*, Colorectal[Title/Abstract])) OR (Colorectal Carcinoma*[Title/Abstract])) OR (Carcinoma*, Colorectal[Title/Abstract])))) AND (("Polymorphism, Single Nucleotide"[Mesh]) OR (((((((((((Single Nucleotide Polymorphism*[Title/Abstract]) OR (Nucleotide Polymorphism*, Single[Title/Abstract])) OR (Polymorphism*, Single Nucleotide[Title/Abstract])) OR (SNP[Title/Abstract])) OR (SNPs[Title/Abstract])) OR (polymorphism*[Title/Abstract])) OR (genetic variant*[Title/Abstract])) OR (variant*[Title/Abstract])) OR (variation*[Title/Abstract])) OR (Nucleotide Polymorphism*[Title/Abstract])) OR (genetic Polymorphism*[Title/Abstract])))) AND (("MicroRNAs"[Mesh]) OR (((((((((((((((((MicroRNA[Title/Abstract]) OR (miRNAs[Title/Abstract])) OR (Micro RNA[Title/Abstract])) OR (RNA, Micro[Title/Abstract])) OR (miRNA[Title/Abstract])) OR (Primary MicroRNA[Title/Abstract])) OR (MicroRNA, Primary[Title/Abstract])) OR (Primary miRNA[Title/Abstract])) OR (miRNA, Primary[Title/Abstract])) OR (pri-miRNA[Title/Abstract])) OR (pri miRNA[Title/Abstract])) OR (RNA, Small Temporal[Title/Abstract])) OR (Temporal RNA, Small[Title/Abstract])) OR (stRNA[Title/Abstract])) OR (Small Temporal RNA[Title/Abstract])) OR (pre-miRNA[Title/Abstract])) OR (pre miRNA[Title/Abstract])))

1. **Web of Science**

(TS=("Colorectal Neoplasm*" OR "Colorectal Tumor*" OR "Colorectal Cancer*" OR "Colorectal Carcinoma*")) AND (TS=("Single Nucleotide Polymorphism*" OR Polymorphism* OR SNP* OR "genetic variant*" OR variant* OR variation* OR "Nucleotide Polymorphism*" OR "genetic Polymorphism*")) AND (TS=(MicroRNA* OR miRNAs OR "Micro RNA" OR miRNA OR "Primary MicroRNA" OR "Primary miRNA" OR "pri-miRNA" OR "pri miRNA" OR "Small Temporal" OR "Temporal RNA" OR stRNA OR "Small Temporal RNA" OR pre-miRNA OR "pre miRNA"))

1. **EMBASE**

#10

#3 AND #6 AND #9

1,002

#9

#7 OR #8

257,215

#8

microrna*:ti,ab,kw OR mirnas:ti,ab,kw OR 'micro rna':ti,ab,kw OR 'rna, micro':ti,ab,kw OR mirna:ti,ab,kw OR 'primary microrna':ti,ab,kw OR 'microrna, primary':ti,ab,kw OR 'primary mirna':ti,ab,kw OR 'mirna, primary':ti,ab,kw OR 'pri mirna':ti,ab,kw OR 'rna, small temporal':ti,ab,kw OR 'temporal rna, small':ti,ab,kw OR strna:ti,ab,kw OR 'small temporal rna':ti,ab,kw OR 'pre mirna':ti,ab,kw

200,909

Edit

Email alert

RSS feed

#7

'microrna'/exp

241,702

#6

#4 OR #5

2,003,600

#5

'single nucleotide polymorphism*':ti,ab,kw OR 'nucleotide polymorphism*, single':ti,ab,kw OR 'polymorphism*, single nucleotide':ti,ab,kw OR snp:ti,ab,kw OR snps:ti,ab,kw OR polymorphism*:ti,ab,kw OR 'genetic variant*':ti,ab,kw OR variant*:ti,ab,kw OR variation*:ti,ab,kw OR 'nucleotide polymorphism*':ti,ab,kw OR 'genetic polymorphism*':ti,ab,kw

1,957,468

#4

'single nucleotide polymorphism'/exp

251,219

#3

#1 OR #2

509,377

#2

'colorectal neoplasm*':ti,ab,kw OR 'neoplasm*, colorectal':ti,ab,kw OR 'colorectal tumor*':ti,ab,kw OR 'tumor*, colorectal':ti,ab,kw OR 'colorectal cancer*':ti,ab,kw OR 'cancer*, colorectal':ti,ab,kw OR 'colorectal carcinoma*':ti,ab,kw OR 'carcinoma*, colorectal':ti,ab,kw

235,513

#1

'colorectal tumor'/exp

1. **Cochrane Library Databases**

#1 MeSH descriptor: [Colorectal Neoplasms] explode all trees 12601

#2 (Colorectal Neoplasm* OR Neoplasm*, Colorectal OR Colorectal Tumor* OR Tumor*, Colorectal OR Colorectal Cancer* OR Cancer*, Colorectal OR Colorectal Carcinoma* OR Carcinoma*, Colorectal):ti,ab,kw (Word variations have been searched) 20379

#3 #1 OR #2 23718

#4 MeSH descriptor: [Polymorphism, Single Nucleotide] explode all trees 2512

#5 (Single Nucleotide Polymorphism* OR Nucleotide Polymorphism*, Single OR Polymorphism*, Single Nucleotide OR SNP OR SNPs OR polymorphism* OR genetic variant* OR variant* OR variation* OR Nucleotide Polymorphism* OR genetic Polymorphism*):ti,ab,kw (Word variations have been searched) 49103

#6 #4 OR #5 49103

#7 MeSH descriptor: [MicroRNAs] explode all trees 548

#8 (MicroRNA* OR miRNAs OR Micro RNA OR RNA, Micro OR miRNA OR Primary MicroRNA OR MicroRNA, Primary OR Primary miRNA OR miRNA, Primary OR pri-miRNA OR pri miRNA OR RNA, Small Temporal OR Temporal RNA, Small OR stRNA OR Small Temporal RNA OR pre-miRNA OR pre miRNA):ti,ab,kw (Word variations have been searched) 1861

#9 #7 OR #8 1861

#10 #3 AND #6 AND #9 1

**Supplementary material 2** Items for quality evaluation of included studies

1. Representativeness of case

Item 1, Selected from population cancer registry (2')

Item 2, Selected from hospital (1')

Item 3, No method of selection described (0')

2. Representativeness of control

Item 4, Population-based (3')

Item 5, Mixed (2')

Item 6, Hospital-based (1')

Item 7, Not described (0')

3. Ascertainment of cancer

Item 8, Histopathologic confirmation (2')

Item 9, By patient medical record (1')

Item 10, Not described (0')

4. Case-control matching

Item 11, Controls matched with cases by age and sex (2')

Item 12, Controls matched with cases only by age or by sex (1')

Item 13, Not matched or not described (0')

5. Genotyping examination

Item 14, Genotyping done blindly and quality control (2')

Item 15, Only genotyping done blindly or quality control (1')

Item 16, Not described (0')

6. HWE

Item 17, HWE in the control group (1')

Item 18, HWD in the control group or not mentioned (0')

7. Total sample size

Item 19, more than 1000 (3')

Item 20, 501-1000 (2')

Item 21, 201 - 500 (1')

Item 22, less than 200 (0')
