# Supplementary material for: Polymorphic Single‐Nucleotide Variants in miRNA Genes and the Susceptibility to Colorectal Cancer: Combined Evaluation by Pairwise and Network Meta‐Analysis, Thakkinstian's Algorithm and FPRP Criterium
Source: Cancer Med. 2025 Jan 22;14(2):e70621. doi: 10.1002/cam4.70621 (PMC11751872; doi:10.1002/cam4.70621)
Supplement: Supplementary file 4 — Data S5. [file CAM4-14-e70621-s002.docx]

**
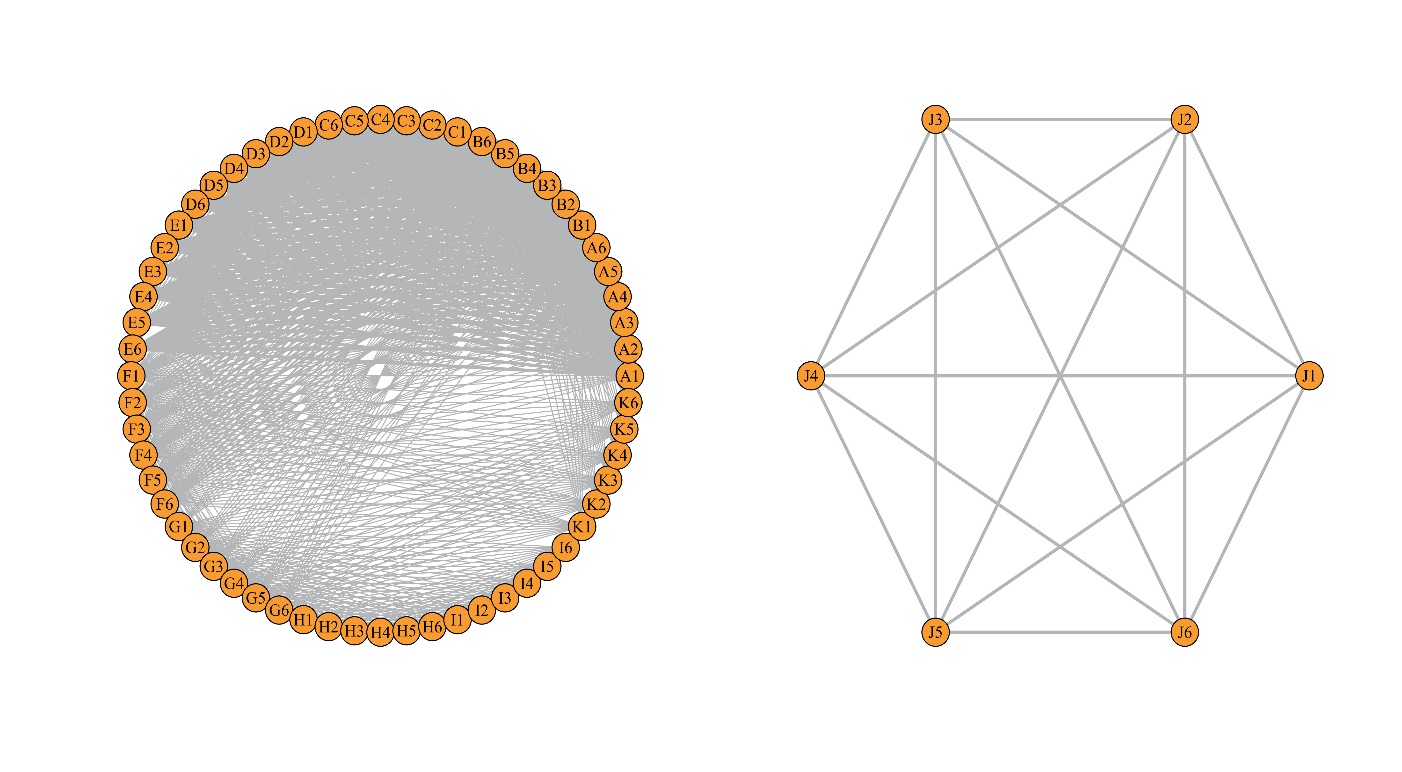
**

**Supplementary material 5** Network meta-analysis results for the genetic models of the CRC risk-related SNPs. **miRNA-SNPs**: (A) miR-196a2 (rs11614913), (B) miR-146a (rs2910164), (C) miR-27a (rs895819), (D) miR-499 (rs3746444), (E) miR-149 (rs2292832), (F) miR-608 (rs4919510), (G) miR-143/145 (rs41291957), (H) miR-143/145 (rs353292), (I) miR-143/145 (rs4705341), (J) miR-34b/c (rs4938723), (K) miR-124 (rs531564). **Genetic models**: (1) allele contrast model, (2) homozygous model, (3) heterozygous model, (4) dominant model, (5) recessive model, and (6) over-dominant model.
